# Nutritional Practices Among Ethnic Minority High School Students in Mountainous Regions, Vietnam

**DOI:** 10.3390/ijerph22071021

**Published:** 2025-06-27

**Authors:** Nhung Thi Ninh, Chinh Thi Kieu Pham, Nga Thi Thanh Nguyen, Tu Thi Thanh Pham, Huong Thi Lan Dao, Lien Phuong Vu, Minh Thi Tran, Quang Van Mai

**Affiliations:** 1 Thai Binh University of Medicine and Pharmacy, Thai Binh 410000, Vietnam; chinhptk@tbump.edu.vn; 2 Tay Bac University, Son La 34000, Vietnam; ngantt@utb.edu.vn (N.T.T.N.); thanhtu.bio@utb.edu.vn (T.T.T.P.); daolanhuong@utb.edu.vn (H.T.L.D.); lienvp@utb.edu.vn (L.P.V.); minhtt@utb.edu.vn (M.T.T.); maivanquang@utb.edu.vn (Q.V.M.)

**Keywords:** malnutrition, adolescents, ethnic minorities, mountainous regions, Vietnam

## Abstract

Background: Vietnam is experiencing a significant “double burden” of malnutrition, characterized by the persistent challenge of undernutrition, particularly stunting in ethnic minority and mountainous regions, alongside a rising prevalence of overweight and obesity. Understanding dual nutritional status and its related factors in adolescents from these vulnerable areas is crucial for effective intervention. Objective: The current study explored the nutritional status of ethnic minority secondary school students in some mountainous provinces in the northwest of Vietnam and identified some related factors. Methods: We conducted a cross-sectional study over six months (June–December 2023), involving 1847 ethnic minority high school students from Son La and Dien Bien provinces, recruited through convenience sampling in primary healthcare settings. Nutritional status was determined using WHO 2006 Child Growth Standards, specifically height-for-age Z-scores (HAZ) and body mass index (BMI)-for-age Z-scores (BAZ). A 4-point scale assessed nutritional practices, including food consumption frequency and eating habits. Results: This study identified a stunting (HAZ < −2 SD) prevalence of 16.7% and wasting (BAZ < −2 SD) of 5.6%; overweight/obesity (BAZ > +1 SD) prevalence was 8.2%. Key factors related to stunting were being male, having more siblings, poor/near-poor household economic status, low maternal education level, and frequent occurrences of gastrointestinal and respiratory diseases. A higher risk of overweight/obesity was associated with consuming more than three meals daily, nocturnal snacking, frequent intake of fried foods, insufficient consumption of vegetables and fruits, and inadequate daily physical activity. Conclusions: Our study shows a comprehensive picture of malnutrition among children in ethnic minority areas. Essential nutritional intervention programs, projects, and models are a top priority to reduce the disease burden for children’s bright future and to enhance socio-economic development in the mountainous regions of Vietnam.

## 1. Introduction

Nutrition is considered the foundation of health; a reasonable diet will create favorable conditions for maximum development in stature, physical strength, and intelligence. Nutritional needs for each stage of the body’s development are different. Adolescence is a critical period of rapid growth and development, making optimal nutrition during these years fundamental for achieving full adult potential and for long-term health. Globally, adolescent malnutrition, encompassing both undernutrition and overnutrition, poses significant public health challenges, impacting physical growth and cognitive development and increasing susceptibility to diseases later in life [1,2].

For high school age (high school students in Vietnam are typically aged 15–18 years), it is an important transition period with many psychological, physiological, and physical changes [3,4]. This is also the country’s future workforce. Many studies suggest that 25% of human height is achieved during adolescence; this is a period of very rapid growth in terms of weight, height, and muscle, as well as fat reserves, so it is necessary to have a good diet and a healthy living environment for children to have the best physical development [5,6]. Vietnam is a multi-ethnic country, with the Kinh (Viet) people constituting the majority of the population. Distinct from the Kinh, there are 53 officially recognized ethnic minority groups, each with unique languages, cultural traditions, and social practices. These groups collectively account for approximately 14% of the national population and are predominantly concentrated in the mountainous and highland regions, particularly in the Northwest, Central Highlands, and Mekong Delta. Historically and presently, many ethnic minority communities face significant socio-economic challenges compared to the Kinh majority. These often include higher rates of poverty, limited access to quality education and healthcare services due to geographical remoteness and sometimes language barriers, lower agricultural productivity on less fertile land, and greater vulnerability to food insecurity. Ethnic minority populations worldwide, and particularly in developing countries like Vietnam, often experience disproportionately higher rates of malnutrition due to a complex interplay of factors. These can include limited access to diverse and nutritious foods, lower socioeconomic status, geographical isolation, distinct cultural food practices, and lower access to health and nutrition education and services [1,2]. These challenges are often exacerbated in mountainous regions, further hindering optimal nutritional outcomes for adolescents. The nutritional census report in Vietnam shows that malnutrition and stunting among school-age children decreased from 23.4% in 2010 to 14.8% in 2020. However, disparities between regions in the rate of stunting are still high, especially in rural and mountainous areas. In addition, the rate of overweight and obesity increased sharply from 8.5% in 2010 to 19% in 2020. Notably, in rural and mountainous areas, this rate also increased significantly [6].

The data from the 2019 nutrition monitoring system shows that stunting malnutrition in mountainous areas accounts for a high rate (38%) [7]. According to a report by the World Bank and the Institute of Nutrition, ethnic minorities in Vietnam often face malnutrition; ethnic minority children have a malnutrition rate twice as high as that of Kinh children (31.4% compared to 15%) and the rate of ethnic minority children with underweight malnutrition is 2.5 times higher than that of children in other regions (21% compared to 8.5%). Furthermore, up to 60% of children with stunting in the 10 provinces with the highest stunting rates in the country are ethnic minorities [7].

Although there have been many studies assessing the prevalence of malnutrition and undernutrition in children under 5 years of age and adults, studies on nutritional status in school-age children, especially children in ethnic minority areas, are still limited. Therefore, this research is crucial for establishing a basis from which to propose timely measures to minimize nutrition-related health problems of school-age students and help propose appropriate and specific intervention solutions for students in disadvantaged areas in the northwestern mountainous province. Our project was conducted to describe the nutritional status and some related factors of ethnic minority high school students in some northwestern mountainous provinces in Vietnam.

## 2. Materials and Methods

### 2.1. Research Subjects

Ethnic minority students aged 15–18 years, studying at the selected high schools in Son La and Dien Bien, agreed to participate in the study. Students with hunchback, scoliosis, and chronic diseases at the time of the study were excluded.

### 2.2. Research Design

A cross-sectional study was conducted on the student population of 6 high schools in 2 provinces, Son La and Dien Bien, located in the mountainous region of Northwest Vietnam from June 2023 to December 2023, including Son La and Dien Bien. Ethnic minorities often concentrate in mountainous and remote areas [8].

### 2.3. Sample Size and Sample Selection

#### 2.3.1. Sample Size Consideration

A formal sample size calculation for a cross-sectional study estimating a proportion could be based on the following formula:

$$n={Z^{2}}_{\left(1-\alpha /2\right)}\frac{p(1-p)}{d^{2}}$$

where

*n* = required sample size;

*Z* = Z-score corresponding to the desired confidence level (1.96 for 95% confidence);

*P* = estimated prevalence of malnutrition (stunting). Based on previous national data in mountainous areas (38% stunting from the 2019 nutrition monitoring system), *P* could be estimated at 0.38.

*d* = desired margin of error (0.05 for 5%). Using these assumptions (*Z* = 1.96, *P* = 0.38, *d* = 0.05), the calculated sample size would be approximately 365 students. To account for a multi-stage sampling design effect (design effect of 1.5–2) and potential non-response, the sample size would need to be adjusted upwards.

Our enrolled sample of 1847 students far exceeds this estimated minimum, providing robust data for the analyses undertaken. Power considerations for detecting differences or associations would typically aim for 80% power (*β* = 0.20).

#### 2.3.2. Sampling Method and Study Population

This study employed a multi-stage convenience sampling method. Each province (Son La and Dien Bien) is divided into three regions: city center, town, and commune of Region III. The six selected regions include Son La city, It Ong town, Co Ma commune (Son La), Dien Bien city, Muong Cha town, and Bung Lao commune (Dien Bien). Within each selected school, all ethnic minority students who met the inclusion criteria and were present during the study period were invited to participate. This convenience approach at the final stage (student selection within schools) was chosen due to logistical constraints in these remote mountainous areas, aiming to maximize participation from the accessible population within the pre-defined geographical clusters. While not a random probability sampling method, the multi-stage approach aimed to capture a diverse representation of students across different socio-geographic settings (city center, town, and remote commune) within the two provinces.

This study focused specifically on ethnic minority students. Within the selected schools, only students identified as belonging to an ethnic minority group and meeting other inclusion criteria were invited to participate. A total of 1847 students participated in the survey, with a response rate of 100%.

### 2.4. Methods and Techniques Applied in Research

- Interview:

Before participating in the interview, the interviewer introduces the participants and their guardians to the purpose of the study. Then, the subjects are instructed and agree to sign the consent form to participate in the study.

Using pre-designed questionnaires and checklists, different information is collected from the research subjects about:

+ General information of the research subjects.
+ Some factors related to nutritional status.
+ Knowledge, attitudes, nutritional practices, and physical activity.

The questionnaire was developed by the research team based on a review of the existing literature, WHO recommendations for nutritional assessment in adolescents, and previous national nutrition surveys. It included sections on socio-demographic information, factors related to nutritional status, and nutritional practices (knowledge, attitudes, food consumption frequency, and physical activity). The questionnaire was pre-tested on a sample of 30 high school students (not included in the final study sample) in a similar mountainous area to assess clarity, comprehensibility, and time taken for completion. Minor revisions were made to question wording and flow based on the pre-test feedback to ensure its suitability for the target population.

- Age calculation method:

The age of students in the study was calculated according to WHO conventions. Data on date of birth and survey data are entered into the WHO Anthroplus software (version 2007; World Health Organization, Geneva, Switzerland) to calculate the child’s age. Age is calculated in 12 months, specifically:

+ Children from 180 months to under 192 months are 15 years old;
+ Children from 192 months to under 204 months are 16 years old;
+ Children from 204 months to under 216 months are 17 years old;
+ Children from 216 months to under 228 months are 18 years old.

- Evaluate anthropometric characteristics

Height and weight are measured according to routine methods prescribed in anthropometric surveys. We used a TANITA scale (Body Composition Analyzer SC—331S; Tanita Corporation, Tokyo, Japan) with an accuracy of 0.1 kg. The weighing device was placed in a stable and flat position with adequate lighting, checked, and calibrated before weighing. We measured standing height using a Seca portable stadiometer (Seca GmbH & Co. KG, Hamburg, Germany) with a minimum scale of 0.1 cm, which was assembled and placed close to a flat wall plane and perpendicular to a stable floor surface. The wall surface must be flat, and the floor surface must be flat and stable.

Assessment of nutritional status: Investigators used Z-scores (standard deviations) of height-for-age and body mass index (BMI)-for-age to assess malnutrition in children aged 5 to 19. Details of the criteria for comparing currently used indicators are shown in Table 1 and Table 2 [9].

#### Assessment of Physical Activity

- Health risk behaviors related to nutritional status: The present study assessed some risk factors associated with nutritional status of ethnic minority high school students. These factors include household economic conditions, the number of children born living in the family, occupation, mother’s education level, respiratory and digestive diseases, and student information, such as physical activity time, sedentary time, total number of meals per day, breakfast and snack habits, and food consumption frequency of students. At least 60 min a day is the amount of time that children and adolescents should participate in moderate to vigorous physical activities, including sports games, activities, recreation, fitness classes and sports, school sports, activities during breaks between classes, walking, running, or cycling to school and extracurricular activities, picnics, practicing sports, etc. [10].
- Food consumption frequency: A 4-point scale (never, monthly/seasonal, weekly, and daily) will be used to assess the frequency of consumption of primary food groups that students use. Food groups are divided into three groups based on macronutrient composition, including (i) carbohydrate group (rice, white bread, instant noodles, vermicelli/noodles/rolls, potatoes, and tubers); (ii) protein group (meat, eggs, fish, shrimp, crab, and animal organs); and (iii) fat group (fat and cooking oil).

In addition, vegetables and fruits, snacks (canned foods, soft drinks, candy, and snacks/chips), and fried foods are also assessed separately to further assess participants’ frequency of consumption.

- Determining household economic status: According to the classification of the Commune People’s Committee according to the Prime Minister’s decision in 2015.

Students’ physical activity levels are determined based on WHO recommendations: children aged 5–19 years old should participate in 60 min of physical activity/day, of which strenuous activities ranging from moderate to severe should be performed at least 2–3 times/week [11].

### 2.5. Statistical Analyses

Data were entered using Epidata software (version 3.1; Epidata Consortium, Odense, Denmark; https://www.epidata.dk (accessed on 23 June 2025)). Anthropometric data were processed and Z-scores for height-for-age (HAZ) and BMI-for-age (BAZ) were calculated using WHO AnthroPlus software (version 2007; World Health Organization, Geneva, Switzerland; https://www.who.int/tools/growth-reference-data-for-5to19-years/application-tools (accessed on 23 June 2025)). Subsequent statistical analyses were performed using SPSS software (version 20.0; IBM Corp., Armonk, NY, USA). Qualitative data are presented as frequencies and percentages. Quantitative variables with normal distribution are presented as mean value ($\bar{X}$) and standard deviation (SD). Children’s weight and height are entered using WHO-Anthro plus 2007 to calculate Z-score BMI/age and height/age. ANOVA test and *T*-test are used to compare average values with normal distribution. Non-parametric tests are used to test mean values that are not normally distributed. The χ2 test is used to compare percentages. The first quartile was used as a reference to estimate odds ratios (OR) and 95% confidence intervals (CI). A two-sided *p* value of less than 0.05 was considered statistically significant. Binary logistic regression analysis was employed to calculate odds ratios (OR) and their 95% confidence intervals (CI) for factors associated with stunting and overweight/obesity. Initially, univariate logistic regression was performed for each potential factor. Factors found to be significant in the univariate analysis (*p* < 0.20) or deemed epidemiologically important were then included in a multivariate logistic regression model using a backward stepwise selection method to identify independent predictors. Covariates considered for adjustment in the multivariate models included gender, age, economic conditions, number of children in the family, mother’s education level, frequency of gastrointestinal/respiratory diseases for stunting, and number of meals/day, snacking habits, fried food consumption, fruit/vegetable intake, and physical activity levels for overweight/obesity, as appropriate.

### 2.6. Ethical Consideration

This study complied with the review process of the Ethics Committee of Tay Bac University (code: B2023-TTB-04; date of approval: 15 June 2023), based on the principles of the Declaration of Helsinki. The data collection process is carried out under the approval of the Department of Education and Training of Son La and Dien Bien. Data is confidential and used for research purposes only. Students participate voluntarily and can withdraw from the study at any time. For participants under 18 years of age, written informed consent was obtained from their parents or legal guardians, and student assent was also secured. For participants aged 18, their own written informed consent was obtained. Subject information is kept confidential and is only used for research.

## 3. Results

### 3.1. General Characteristics of Study Participants by Gender

Students are Thai ethnic group (48.6%), Mong ethnic group (46.1%), and other ethnic (Tay, Muong, and Khang) groups (5.3%). The average age is 17.02 ± 0.84. A total of 38% are from poor/near-poor households. Regarding anthropometric characteristics, the average height and weight of male students are 161.3 ± 6.9 cm and 51.9 ± 8.8 kg and of female students are 155.2 ± 6.2 cm and 47.8 ± 7.6 kg. The average HAZ index is −1.27 ± 0.88, higher in females than in males (*p* < 0.05) (Table 3).

### 3.2. Nutritional Status

The prevalence of both severe stunting (5.2% vs. 3%) and moderate stunting (13.8% vs. 11%) was higher in male students compared to female students (Table 4). In addition, the rate of wasting in male students according to BMI for age was higher than that in female students (6.2% and 5%, respectively). The rate of overweight/obesity in ethnic minority students was 8.2%.

### 3.3. Health Risk Behaviors Related to Nutritional Status

In Table 5, health risk behaviors related to nutritional status of ethnic minority school-age students are shown. About 19.9% of students regularly skipped breakfast, 21.2% only eat two meals/day, 19.5% eat snacks after dinner, 36.5% regularly eat fried foods, 15.9% often eat snacks, and 75.9% and 61.7% regularly eat green vegetables and fruits. In total, 29.7% met recommendations for physical activity and the average physical activity time was 42.8 ± 25.8 min/day, with differences between men and women.

Table 6 shows factors related to stunting in male students (OR = 1.4; 95% CI: 1.1–1.9), living in a family with more than two children (OR = 1.9; 95% CI: 1.5–2.5), poor or near-poor households (OR = 3.0; 95% CI: 2.3–3.8), low maternal education level (OR = 1.9; 95% CI: 1.5–2.4), frequent digestive diseases (OR = 3.5; 95% CI: 2.6–4.6) and respiratory diseases (OR = 3.3; 95% CI: 2.5–4.4).

The results in Table 7 found several factors associated with overweight and obesity among students eating more than three meals/day (OR = 2.8; 95% CI: 1.9–3.9); eating extra snacks in the evening (OR = 2.4; 95% CI: 1.7–3.5); frequently eating fried foods (OR = 2.4; 95% CI: 1.7–3.3); frequent snacking (OR = 3.0; 95% CI: 2.1–4.3); rarely/sometimes eating vegetables and fruits (OR = 1.7; 95% CI: 1.2–2.4); and physical activity not meeting needs (OR = 3.2; 95% CI: 1.9–5.2).

## 4. Discussion

This study contributes novel data by focusing specifically on the double burden of malnutrition among diverse ethnic minority adolescents in the underserved northwestern mountainous region of Vietnam, an area often underrepresented in national surveys, providing crucial region-specific insights. The current study is one of the few studies in Vietnam showing the double burden of malnutrition among ethnic minority high school students living in the northwest mountainous region, of which the rate of stunting malnutrition is 16.7%, higher than the results of the Vietnam Nutrition Survey 2019–2020 [5], with the rate of stunting malnutrition in school-age children (5–19 years old) being 14.8%. The main reason for this difference is the difference in characteristics of the northwest mountainous region of Vietnam compared to the provinces and cities in the plains, specifically, the terrain is mainly mountainous–plateau, the household economy is mainly agricultural, and living conditions are difficult and do not guarantee basic needs. Stunting at this stage is the consequence of prolonged malnutrition in many previous stages, the consequences of which are difficult to recover because the majority of adolescents are 15–18 years old.

Compared with the study of author Hoang Van Phuong on the same subjects, our results showed a lower rate [12]. Each ethnic minority has its characteristics in terms of physical appearance, socio-economic conditions, customs, and living standards, which have different effects on nutritional status. At the same time, our evaluation time is that after 4–6 years, economic and intellectual conditions have improved, and nutrition programs have been somewhat effective, so the malnutrition rate has improved. However, it is still much higher than in the delta areas and big cities as researched by Nguyen Hoa (10.8%) [13], Le Tran Tuan Anh (6.4%) [14], and Nguyen Thi Trung Thu (3.9%) [15]. The above results once again show that differences in economic, social, and living conditions have a profound impact on nutritional status.

Along with the problem of malnutrition, there is an alarming increase in overweight and obesity rates among children and adolescents. The overall prevalence of overweight and obesity in our study was 8.2%, lower than the overall prevalence of overweight and obesity in the 2020 institute survey study of 19% [6]. However, it is 6.9% higher than in mountainous areas [6], lower than research results in the delta such as research by Nguyen Hoa (9.6%) [13] and Ngo Hong Nhung (13.8%) [16] and lower than some countries such as Northeast Brazil (34.5%) [17] and Poland 25% [18]. Vietnam is experiencing the double burden of nutrition: undernutrition in disadvantaged areas and overweight/obesity in urban areas. However, overweight and obesity are also gradually appearing in several children in rural and mountainous areas due to inadequate and unbalanced nutrition. Therefore, we still need proper attention and intervention to minimize the increase in this condition.

The basic causes of malnutrition are poverty, socio-economic disadvantages, and economic inequality. Ethnic minorities reside mainly in the Northwest, Central Highlands, Southwest, and Central Coast and account for 13.44% of the country’s population but account for 52.7% of the country’s poor [19]. The current study shows that students with poor or near-poor economic conditions are at risk of stunting and wasting is higher than in students from families with average/good economic conditions. It can be seen that the above statements are very consistent with the UNICEF model of causes and consequences of malnutrition and previous reports [20]. Nguyen Thi Loan and colleagues showed that students in poor/near-poor families are twice as likely to have rickets as students from better-off families [21]. The explanation for this may be that children born in better socio-economic conditions will have better medical care and education, leading to better nutritional practices, receiving care and guidance on a more reasonable diet, thereby giving birth to healthy children, creating a future generation that develops well both physically and mentally.

This study also shows that stunting and wasting are higher in male students than in female students. This result is similar to Nguyen Van Tam [22] and Truong Thi Thu Huong [23]. The differences between studies may be due to the different developmental stages of boys and girls, specifically, there are differences in living habits, eating habits, resistance, common diseases, and physiological development speed. On the other hand, boys are often more vulnerable and have a higher risk of disease than girls. At the same time, during this period, girls pay more attention to physical awareness, leading to proper nutrition and physical activity to achieve good nutritional status.

Regarding socio-economic conditions, our research has shown that children born and raised in families with two or more children are at higher risk of malnutrition. The prevalence of large families in ethnic minority areas is a well-documented and persistent reality, influenced by local customs and beliefs. People talk about having children, having a housekeeper or having a son to worship, continuing the family line, and early marriage. The situation of having too many children leads to a series of consequences such as economic difficulties, difficulty raising children, and, at the same time, women’s health declines. It has a significant impact on social life. Many households need help ensuring nutritious meals for their children. In addition, they face household food insecurity, which is thought to be associated with malnutrition due to low food quality and quantity, adding to the heavy burden of malnutrition in children [8].

The mother’s education level is one factor that affects students’ nutritional status. For mothers with higher education levels, the rate of stunting and wasting in children is lower. This is similar to the study by author Nguyen Thi Loan, which also shows that students whose mothers have an education level of less than secondary school have a 1.6 times higher risk of stunting than students whose mothers have an education level of lower secondary school or higher (*p* < 0.05) [21]. Research on 12–18-year-old children in Nigeria shows that age and household economy are related to stunting [24]. This association is explained by the fact that mothers with higher levels of education tend to have better knowledge and practices about child nutrition. In addition, mothers with higher levels of education have access to more information sources that help supplement their understanding of child-rearing and care more scientifically and methodically.

Malnutrition and respiratory and digestive diseases such as pneumonia, diarrhea, loose stools, or constipation are often closely related. Illness in children leads to anorexia, reduced absorption of nutrients, and limited food intake, leading to malnutrition. When children suffer from respiratory and digestive tract infections, their need for energy and nutrients increases. However, given that many mothers in these communities have lower levels of education, they often adhere to traditional concepts of dietary restriction during illness, for example, they do not let children eat greasy foods when they have loose stools or diarrhea and do not feed fishy foods according to folk beliefs. However, after the child recovers, not feeding them to compensate for the previous period leads to malnutrition, which is common in these children. In our study, students that frequently suffered from gastrointestinal diseases and respiratory diseases have a higher risk of stunting and malnutrition when having frequent gastrointestinal diseases and respiratory diseases.

Students’ eating habits are an important factor affecting nutritional status. Our research shows that students who eat more than three meals/day and eat an extra meal in the evening have a higher risk of being overweight and obese. Many lifestyle studies show that, when consuming food late at night, the body is more likely to cause excess energy and fat will accumulate in the body, causing weight gain [25,26]. Our study shows that students who regularly eat fried foods and snack foods are at risk of being overweight and obese. This is perfectly reasonable because fried foods often contain significantly more fat and calories than non-fried foods prepared by other methods. On the other hand, toxic substances will appear during processing at high temperatures, affecting health. Moreover, the palatability, variety, and convenience of processed snacks have increased student demand for these foods. Our study found a relationship between snacking habits and overweight and obesity; specifically, students who regularly snack have a higher risk of being overweight and obese compared with other students. The study by Aljefree et al. also showed that obese students consumed potato chips (56.8% vs. 45.2%; *p* = 0.004), popcorn (41.7% vs. 33.3%; *p* = 0.04), and cookies (20.9% vs. 14.3%; *p* = 0.04) more frequently than non-obese students [27].

In addition, the current study showed that students who rarely or occasionally eat vegetables and fruits have a higher risk of being overweight and obese (OR = 1.7; 95% CI: 1.2–2.4). Similar to the study by author Le Thi Huong, students who do not eat vegetables regularly have a 1.57 times higher risk of being overweight and obese [28]. The study by author Le Huy Hoang and colleagues also showed that the risk of being overweight and obese in children is reduced when children regularly eat fruits and regularly eat green vegetables (*p* < 0.05) [27]. According to the 2020 Vietnam Nutrition Survey report, the average amount of vegetables and fruits consumed per capita has increased from 190.4 g of vegetables/person/day and 60.9 g of ripe fruit/person/day (2010) to 231 g of vegetables/person/day and 140.7 g of ripe fruit/person/day (2020). However, the amount of vegetables and fruits consumed was about 66.4–77.4% of the recommended needs [8]. Meanwhile, a diet rich in meat cannot provide enough fiber needed for a healthy digestive system and not enough vitamins for comprehensive development. Therefore, parents and caregivers need to increase their diet with vitamins and green vegetables and limit and reduce fatty foods, especially fast food and food at convenience stores.

Not only nutrition but also physical activity contributes significantly to the comprehensive development of students. Exercise will help children be healthier and more balanced because these activities help children feel mentally comfortable and have a better appetite. However, students who do not exercise or do not participate in physical activities will not burn off all their energy, leading to overweight and obesity. Students who exercise less than 8 h/day and whose exercise time does not meet the recommended needs are at higher risk of being overweight and obese. Ngo Thi Xuan et al. have shown that students who exercised less in the previous week (time spent watching TV, using computers/surfing the web, taking extra classes, and reading books/stories) have a higher rate of overweight and obesity than other groups of students [29]. On the other hand, in a study of primary school children in Pakistan, children who participated in vigorous physical activity more than twice a week had a lower risk of being overweight or obese (OR = 0.49), similar to children with a sedentary lifestyle > 1 h/day, which increased the risk of being overweight and obese by 1.56 times compared to other children [30].

These results provide important evidence to develop long-term interventions to improve child nutrition nationwide, especially for adolescents from ethnic minorities.

### 4.1. The Current Study Draws Some Implications

Firstly, it is necessary to organize nutrition education communication sessions with the participation of parents and caregivers of students and distribute leaflets with instructions on proper nutrition to households. Parents should be instructed on a balanced and reasonable diet and increase the use of foods suitable for the child’s age. In addition, schools should organize extracurricular activities to provide children with information on the harmful effects of malnutrition and simple and easy-to-apply ways to prevent malnutrition and encourage children to practice every day and organize appropriate physical exercise and sports sessions for children. At the same time, parents should be equipped with knowledge about nutrition suitable for the child’s age and spend time preparing appropriate meals for children. Second, families need to ensure food security for impoverished and near-poor households, improve nutrition for children in remote areas, and take advantage of locally available food sources. Third, ethnic minority students should be ensured access to nutritious food and the government’s milk program to improve their weight and height. Finally, it is necessary to guide students to develop and manage positive lifestyle changes, such as monitoring anthropometric indicators, creating a healthy diet, regularly eating vegetables and foods rich in vitamin A, reducing snacking, and increasing physical activity as recommended.

### 4.2. This Study Has Some Limitations

First, the study design was cross-sectional, so it is difficult to establish a causal relationship between undernutrition/overnutrition and to accurately identify risk factors. On the other hand, our study assessed dietary habits based on food consumption frequency but did not investigate the actual diet of students. In addition, recall bias may occur due to students’ responses. The convenience sampling method, while practical for the setting, may limit the generalizability of findings to all ethnic minority adolescents in the northwest region. Furthermore, while we assessed food consumption frequency, a quantitative dietary assessment (24 h recall or food frequency questionnaire with portion sizes) was not conducted, which limits our ability to assess actual nutrient intake. Moreover, the classification of household economic status was based on official commune lists, which might not fully capture the nuances of household wealth or access to resources. Finally, this study did not account for seasonal variations. The data were collected from June to December, a period that spans from the rainy season to the dry season in Northwest Vietnam. Seasonal changes can significantly affect the availability and diversity of local foods, such as agricultural products and wild-harvested vegetables or fruits, which, in turn, influences nutritional intake. Future longitudinal studies should consider assessing nutritional status across different seasons to capture a more dynamic picture of dietary patterns in these communities.

The findings of this study have several important policy implications. Locally, for Son La, Dien Bien, and similar northwestern mountainous provinces, these results highlight the urgent need for targeted, culturally sensitive nutritional interventions. Health policies should prioritize programs that address both undernutrition (stunting at 16.7%) and the emerging issue of overweight/obesity (8.2%). Specific local interventions could include:

- Strengthening school-based nutrition education programs, incorporating culturally appropriate content and involving parents, particularly mothers, given the link between maternal education and stunting.
- Developing and promoting diverse local food systems to improve access to and affordability of nutrient-rich foods, moving beyond reliance on a few staple crops.
- Integrating regular nutrition screening (height, weight, and BMI assessment) and counseling into primary healthcare services and school health programs for early detection and management of malnutrition.
- Implementing community-based programs to improve awareness of healthy eating habits, dangers of frequent consumption of fried foods and processed snacks (associated with ORs of 2.4 and 3.0 for overweight/obesity, respectively), and the importance of regular physical activity. Globally, our findings contribute to the body of evidence on the double burden of malnutrition among adolescents in low- and middle-income countries, particularly within ethnic minority groups. This underscores the need for international health organizations and national governments to advocate for and support equity-focused nutrition policies that specifically target vulnerable and marginalized populations, like ethnic minorities in remote regions; invest in research to better understand the unique determinants of malnutrition in diverse cultural contexts to tailor interventions effectively; and promote multi-sectoral approaches that address the underlying socioeconomic determinants of malnutrition, such as poverty (associated with an OR of 3.0 for stunting), education, and food security, beyond solely health-focused interventions.

## 5. Conclusions

In summary, this study shows that malnutrition is still common among ethnic minority school children in the mountainous northwest of Vietnam, along with the increasing rate of overweight and obesity. These issues are all related to students’ socio-economic conditions, eating habits and living habits. Therefore, it is necessary to develop and deploy nutrition and health education interventions suitable to local culture and ethnic characteristics to attract the community, raise awareness, and practice nutrition. Thanks to that, we can improve the nutritional status of ethnic minority children.

## Figures and Tables

**Table 1:** Evaluation of height-for-age Z-score (HAZ).

Height/Age Z-Score (HAZ)	Evaluate
<−3 SD	Severe stunting malnutrition
<−2 SD	Moderate stunting malnutrition
−2 SD ≤ Zscore ≤ 2 SD	Normal
Z-score > 2 SD	Taller than average

HAZ, height-for-age z-score; SD, standard deviation.

**Table 2:** BMI assessment by age.

Z-Score Index	Z-Score Evaluation
<−3 SD	Severe malnutrition and wasting
−3 SD ≤ Z-score < −2 SD	Wasting
−2 SD ≤ Z-score ≤ 1 SD	Normal
1 SD < Z-score ≤ 2 SD	Overweight
Z-score > 2 SD	Obese

BMI, body mass index; SD, standard deviation.

**Table 3:** Demographic and nutritional characteristics of participants by gender.

Variable		Male *n* (%)	Female *n* (%)	Total *n* (%)	*p*
Class					<0.01 ^1^
	Grade 10	316 (31.9)	318 (37.1)	634 (34.3)	
	Grade 11	378 (38.1)	244 (28.5)	622 (33.7)	
	Grade 12	297 (30.0)	294 (34.4)	591 (32.0)	
Ethnic groups					0.145 ^1^
	Mong	478 (48.2)	374 (43.7)	852 (46.1)	
	Thai	464 (46.9)	434 (50.7)	898 (48.6)	
	Other	49 (4.9)	48 (5.6)	97 (5.3)	
Economic conditions					0.034 ^1^
	Poor households	215 (21.7)	149 (17.4)	364 (19.7)	
	Near poverty	191 (19.3)	146 (17.1)	337 (18.3)	
	Medium	513 (51.7)	492 (57.4)	1005 (54.4)	
	Wealthier	72 (7.3)	69 (8.1)	141 (7.6)	
The number of children born living in the family					0.055 ^1^
	≤2 children	419 (42.3)	400 (46.7)	819 (44.3)	
	>2 children	572(57.7)	456 (53.3)	1028 (55.7)	
Age (years)		17.03 ± 0.82	17.01 ± 0.86	17.02 ± 0.84	0.52 ^3^
Weight (kg)		51.9 ± 8.8	47.8 ± 7.6	50.0 ± 8.5	<0.01 ^2^
Height (cm)		161.3 ± 6.9	155.2 ± 6.2	158.5 ± 7.3	<0.01 ^3^
HAZ		−1.44 ± 0.81	−1.06 ± 0.91	−1.27 ± 0.88	<0.01 ^2^
BAZ		−0.42 ± 1.05	−0.43 ± 0.98	−0.43 ± 1.02	0.825 ^2^

HAZ, height-for-age z-score; BAZ, body mass index-for-age z-score; ^1^ χ2 test; ^2^ Mann–Whitney test; ^3^ *t*-test.

**Table 4:** The distribution of nutritional status of participants by gender.

Nutritional Status		Male *n* (%)	Female *n* (%)	Total *n* (%)	*p*
Stunting malnutrition (height for age)					0.008 ^1^
	Severity level	52 (5.2)	26 (3,0)	78 (4.2)	
	Moderate level	137 (13.8)	94 (11.0)	231 (12.5)	
BMI for age					0.145 ^1^
	Wasting	61 (6.2)	43 (5.0)	104 (5.6)	
	Normal	848 (85.5)	743 (86.8)	1591 (86.1)	
	Overweight/Obese	82 (8.3)	70 (8.2)	152 (8.2)	

^1^ χ2 test; BMI, body mass index.

**Table 5:** The distribution of health risk behaviors of participants by gender.

Health Risk Behavior		Male *n* (%)	Female *n* (%)	Total *n* (%)	*p*
Often do not eat breakfast		215(21.7)	152(17.8)	367(19.9)	0.034 ^1^
Number of meals/day					0.07 ^1^
	2 meals	237 (23.9)	154 (18.0)	391 (21.2)	
	3 meals	538 (54.3)	510 (59.6)	1048 (56.7)	
	>3 meals	216 (21.8)	192 (22.4)	408 (22.1)	
Eat a snack					0.31 ^1^
	Don’t eat snacks	728 (73.5)	661 (77.2)	1389 (75.2)	
	Eat after breakfast	35 (3.5)	24 (2.8)	59 (3.2)	
	Eat after lunch	22 (2.2)	17 (2.0)	39 (2.1)	
	Eat after dinner	206 (20.8)	154 (18.0)	360 (19.5)	
Frequently eat fried foods		352 (35.5)	322 (37.6)	674 (36.5)	0.351 ^1^
Snack often		151 (15,2)	142 (16.6)	293 (15.9)	0.428 ^1^
Eat green vegetables regularly		719 (72.6)	694 (81.1)	1413 (76.5)	<0.01 ^1^
Eat fruit regularly		578 (58.3)	561 (65.5)	1139 (61.7)	<0.01 ^1^
Physical activity					0.471 ^1^
	No	101 (10.2)	95 (11.1)	196 (10.6)	
	Not meeting recommendations	584 (58.9)	518 (60.5)	1102 (59.7)	
	Meets recommendations	306 (30.9)	243 (28.4)	549 (29.7)	
Physical activity time (minutes/day)		5 2.7 ± 26.3	38.9 ±25.4	42.8 ± 25.8	<0.01 ^2^
Sedentary behavior time (minutes/day)		12 4.8 ± 72.4	112.7 ± 74.5	117.3 ±73.1	<0.01 ^2^

^1^ χ2 test; ^2^ Mann–Whitney test.

**Table 6:** Some factors associated with malnutrition and stunting of participants.

Factor		Stunting Malnutrition		OR 95% CI
		Yes *n* (%)	No *n* (%)	
Gender				
	Male	189 (19.1)	802 (80.9)	1.4 * (1.1–1.9)
	Female	120 (14.0)	736 (86.0)	
The number of children born living in the family				
	>2 children	211 (20.5)	817 (79.5)	1.9 * (1.5–2.5)
	≤2 children	98 (12.0)	721 (88.0)	
Ethnic groups				
	Mong	127 (17.4)	604 (82.6)	1
	Thai	172 (16.9)	844 (83.1)	1.03 (0.8–1.3)
	Other	10 (10.0)	90 (90.0)	1.9 (0.96–3.74)
Economic conditions				
	Poor, near-poor	193 (26.0)	549 (74.0)	3.0 * (2.3–3.8)
	Average, well-off	116 (10.5)	989 (89.5)	
Mother’s education level				
	Illiterate, primary school	136 (23.1)	452 (76.9)	1.9 * (1.5–2.4)
	From middle school and up	173 (13.7)	1086 (86.3)	
Frequently suffers from gastrointestinal diseases				
	Yes	91 (35.5)	165 (64.5)	3.5 * (2.6–4.6)
	No	218 (13.7)	1373 (86.3)	
Frequently suffers from respiratory diseases				
	Yes	110 (33.3)	220 (66.7)	3.3 * (2.5–4.4)
	No	199 (13.1)	1318 (86.9)	

* *p* < 0.05.

**Table 7:** Some factors associated with overweight and obesity of participants.

Nutritional Status		Overweight/Obese		OR 95% CI
		Yes *n* (%)	No *n* (%)	
Number of meals/day				
	Over 3 meals	63 (15.4)	345 (85.6)	2.8 * (1.9–3.9)
	≤3 meals	89 (6.2)	1350 (93.8)	
Eat an evening snack				
	Yes	53 (14.7)	307 (85.3)	2.4 * (1.7–3.5)
	No	99 (6.7)	1388 (93.3)	
Frequently eat fried foods				
	Yes	85 (12.6)	589 (87.4)	2.4 * (1.7–3.3)
	No	67 (5.7)	1106 (94.3)	
Snack often				
	Yes	51 (17.4)	242 (82.6)	3.0 * (2.1–4.3)
	No	101 (6.5)	1453 (93.5)	
Eat green vegetables and fruits				
	Rarely/occasionally	50 (11.5)	384 (88.5)	1.7 * (1.2–2.4)
	Frequent	102 (7.2)	1311 (92.8)	
Physical activity meets the recommended needs				
	No	133 (10.2)	1165 (89.8)	3.2 * (1.9–5.2)
	Yes	19 (3.5)	530 (96.5)	

* *p* < 0.05.

## Data Availability

The data that support the findings of this study are available on request from the corresponding author. The data are not publicly available due to privacy or ethical restrictions.

## Abbreviations

The following abbreviations are used in this manuscript:

BAZ	Body Mass Index-for-age z-score
BMI	Body Mass Index
NCHS	National Centre of Health Statistics
HAZ	Height-for-age z-score
SD	Standard Deviation
WHO	World Health Organization
